# Synthesis of [7-^15^N]-GTPs for RNA structure and dynamics by NMR spectroscopy

**DOI:** 10.1007/s00706-022-02892-1

**Published:** 2022-02-26

**Authors:** Kehinde M. Taiwo, Lukasz T. Olenginski, Felix Nußbaumer, Hyeyeon Nam, Stefan Hilber, Christoph Kreutz, T. Kwaku Dayie

**Affiliations:** 1 Department of Chemistry and Biochemistry, Center for Biomolecular Structure and Organization, University of Maryland, College Park, MD 20742 USA; 2 Institute of Organic Chemistry and Center for Molecular Biosciences Innsbruck, University of Innsbruck, Innrain 80/82, 6020 Innsbruck, Austria; 3 Present Address: Center for Cancer Research, National Cancer Institute, Frederick, MD 21702 USA

**Keywords:** Nucleic acids, Isotopic labeling, Spectroscopy, Dynamics

## Abstract

Several isotope-labeling strategies have been developed for the study of RNA by nuclear magnetic resonance (NMR) spectroscopy. Here, we report a combined chemical and enzymatic synthesis of [7-^15^N]-guanosine-5′-triphosphates for incorporation into RNA via T7 RNA polymerase-based in vitro transcription. We showcase the utility of these labels to probe both structure and dynamics in two biologically important RNAs.

**Graphical abstract:**

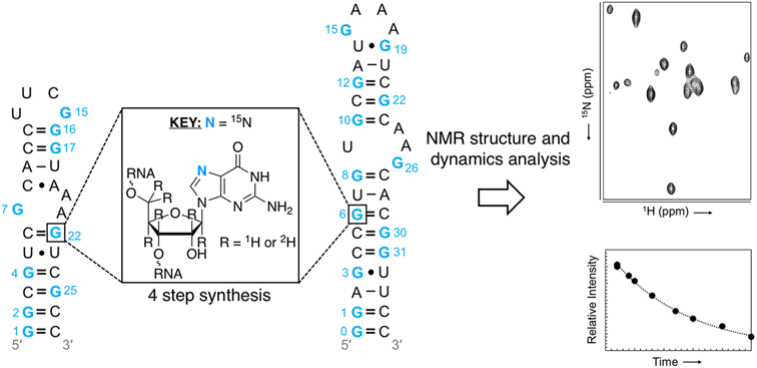

**Supplementary Information:**

The online version contains supplementary material available at 10.1007/s00706-022-02892-1.

## Introduction

RNAs, once thought of as an intermediate in the flow of genetic information from DNA to proteins, are now credited with playing a central role in many cellular functions [1–4]. As a result, RNAs have increasingly become the target of structural and therapeutic efforts [5–7]. Among the different techniques available to study RNA, nuclear magnetic resonance (NMR) spectroscopy is particularly useful [8, 9]. NMR, unlike X-ray crystallography, permits the study of RNA structure and dynamics in solution on a wide-range of timescales spanning picoseconds-to-seconds [10]. Nevertheless, poor chemical shift dispersion and broad linewidths limit the broad application of NMR to understand the structure and dynamics of RNA [11].

Several technological advances have begun to address these problems: use of cryogenic probes, higher magnetic field spectrometers, and the design of multidimensional NMR experiments [12–17]. Even with these innovations, spectral crowding and signal overlap still limit the effective analysis of large (> 30 nucleotide, nt) RNAs [18]. To tackle this problem, we and others have developed labeling technologies to incorporate stable isotopes (i.e., ^2^H, ^13^C, ^19^F, and ^15^N) into RNA to benefit NMR structure and dynamics measurements [7, 19–27].

As part of a larger effort to create atom-specifically labeled ribonucleoside-5′-triphosphates (rNTPs) [7, 20–27], we present the chemical synthesis of [7-^15^N]-guanine from commercially available and inexpensive sodium ^15^N-nitrite. Then, we used enzymes from the nucleotide salvage biosynthetic pathway to couple the labeled nucleobase to commercially available ribose sources to build the corresponding [7-^15^N]-guanosine-5′-triphosphates (GTPs) [20, 28, 29]. This labeling scheme leverages the narrow linewidths of the ^15^N nuclei [30, 31] and will therefore be important when applied to large RNAs. Moreover, the N7 atom in the major groove can serve as a reporter of binding events between nucleic acids and ligands such as metals, drugs, or protein side chains [32, 33].

To showcase the utility of our labels, we incorporated our [7-^15^N]-GTPs into two RNAs [33, 34] via T7 RNA polymerase (RNAP)-based in vitro transcription and used one (1D)-, two (2D)-, and three (3D)-dimensional NMR experiments to probe RNA structure and dynamics.

## Results and discussion

### Synthesis

Synthesis of the isotope-labeled nucleobase was carried out in three chemical steps following previously established methods [35] with slight modifications (Scheme 1). The synthesis of [7-^15^N]-guanine began with the nitrosylation of commercially available 2,6-diaminopyrimidin-4-ol by ^15^N-labeled sodium nitrite to yield 2,6-diamino-5-[nitroso-^15^N]pyrimidin-4-ol (**1**). Reduction by sodium dithionite followed by acidification by sulfuric acid then gave the sulfate salt of 2,6-diamino-5-[amino-^15^N]pyrimidin-4-ol (**2**). In the final chemical step, reflux of **2** with formamide followed by formic acid provided the desired [7-^15^N]-guanine (**3**). Intermediate compounds **1** and **2** showed the expected ^1^H NMR spectra (Figs. S1, S2). The formation and purity of **3** was confirmed by ^1^H, ^13^C, and ^15^N NMR (Figs. S3, S4) and high-resolution mass spectroscopy (HR-MS) (Fig. S5).

**Figure:**
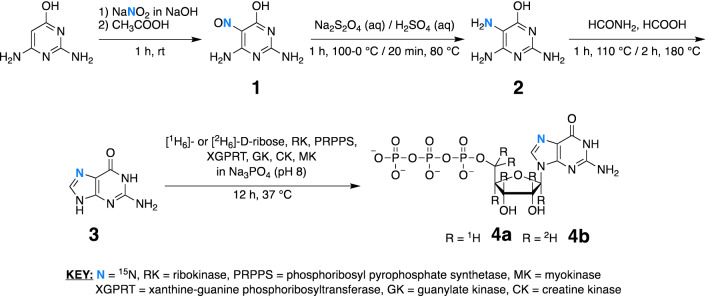

With the labeled nucleobase **3** in-hand, we utilized enzymes from the nucleotide salvage biosynthetic pathway to couple **3** to either fully protonated [^1^H_6_]-d-ribose or fully deuterated [^2^H_6_]-d-ribose to form the desired GTPs **4a** and **4b**, respectively (Scheme 1), as previously described [20]. Briefly, [^1^H_6_]- and [^2^H_6_]-d-ribose were phosphorylated to ribose-5-phosphate (R5P) by ribokinase (RK, EC 2.7.1.15). Then, R5P was converted to 5-phospho-d-ribosyl-*α*-1-pyrophosphate (5PRP) by phosphoribosyl pyrophosphate synthetase (PRPPS, EC 2.7.6.1). Coupling of **3** to 5PRP from the previous step was afforded by xanthine-guanine phosphoribosyltransferases (XGPRT, EC 2.4.2.22) to yield [7-^15^N]-guanosine-5′-monophosphate ([7-^15^N]-GMP). Guanylate kinase (GK, EC 2.7.4.8) converted GMP to [7-^15^N]-guanosine-5′-diphosphate ([7-^15^N]-GDP), which was then phosphorylated by creatine kinase (CK, EC 2.7.3.2) to form the desired GTP products **4a** and **4b**. Complete conversion of **3** to **4a** and **4b** was confirmed by ^31^P NMR (Fig. S6), as previously described [36].

Taken together, our synthetic route provides **4a** and **4b** in three chemical steps and one enzymatic step with a single chromatographic purification. Furthermore, while the present work used [^1^H_6_]- and [^2^H_6_]-d-ribose, our method enables the coupling of **3** to any ribose source to create a versatile assortment of atom-specifically labeled rNTPs for use in in vitro transcription and NMR.

### NMR characterization of atom-specifically labeled RNA

**Fig. 1:**
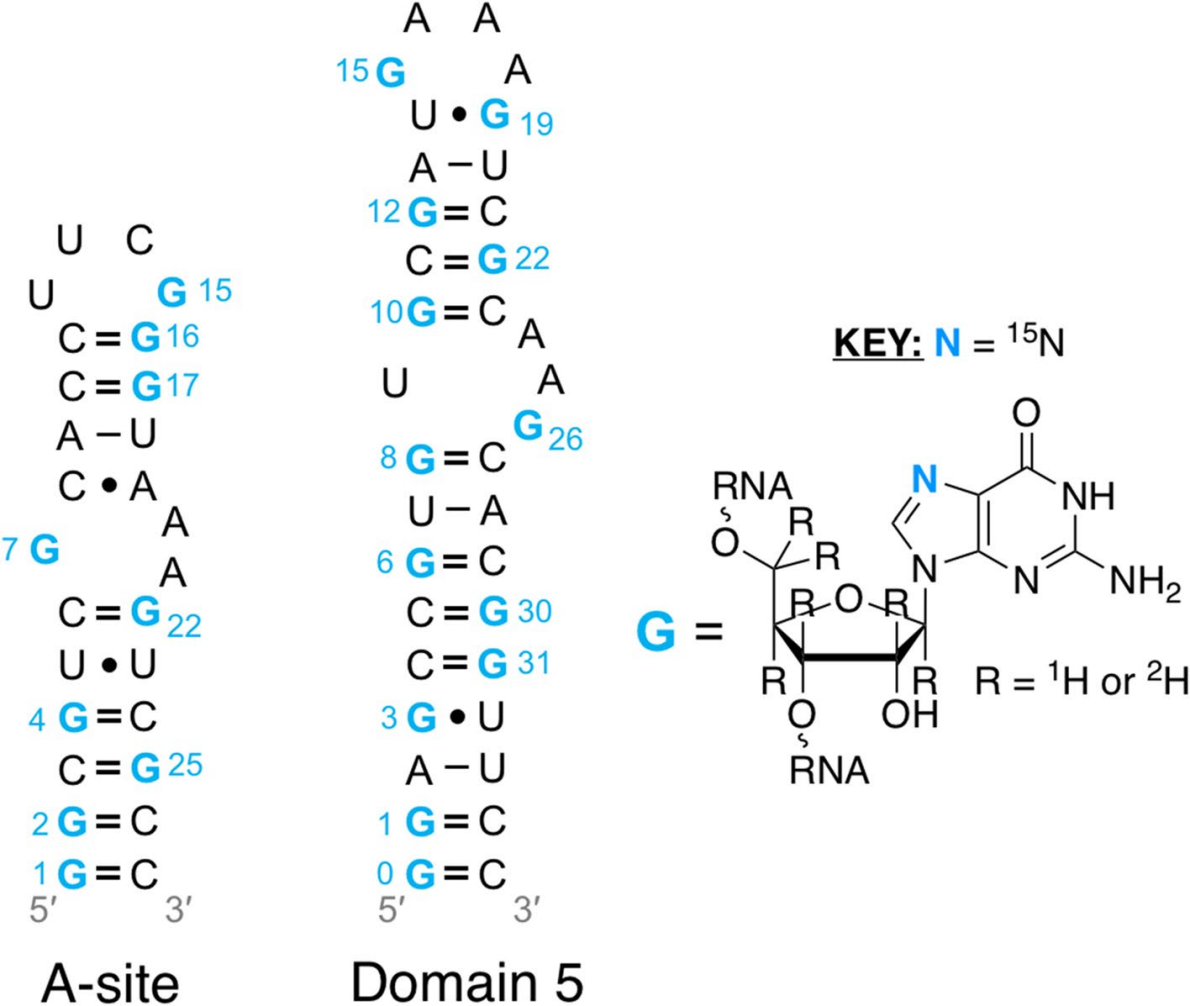
Secondary structure of the 27 nt A-site and 35 nt domain 5 RNAs made from in vitro transcription with **4b** and **4a** incorporated, respectively. Nucleotides labeled with **4a** and **4b** are numbered and shown in blue

Our reason for synthesizing **4a** and **4b** was to characterize the structure and dynamics of biologically important RNAs. Thus, we used **4a** and **4b** along with unlabeled ATP, CTP, and UTP to make two RNAs by in vitro transcription: a **4b**-labeled 27 nt fragment from the human cytoplasmic A-site ribosomal RNA (A-site) and a **4a**-labeled 35 nt fragment from domain 5 of the group II intron ribozyme from brown algae (domain 5) (Fig. 1).

**Fig. 2:**
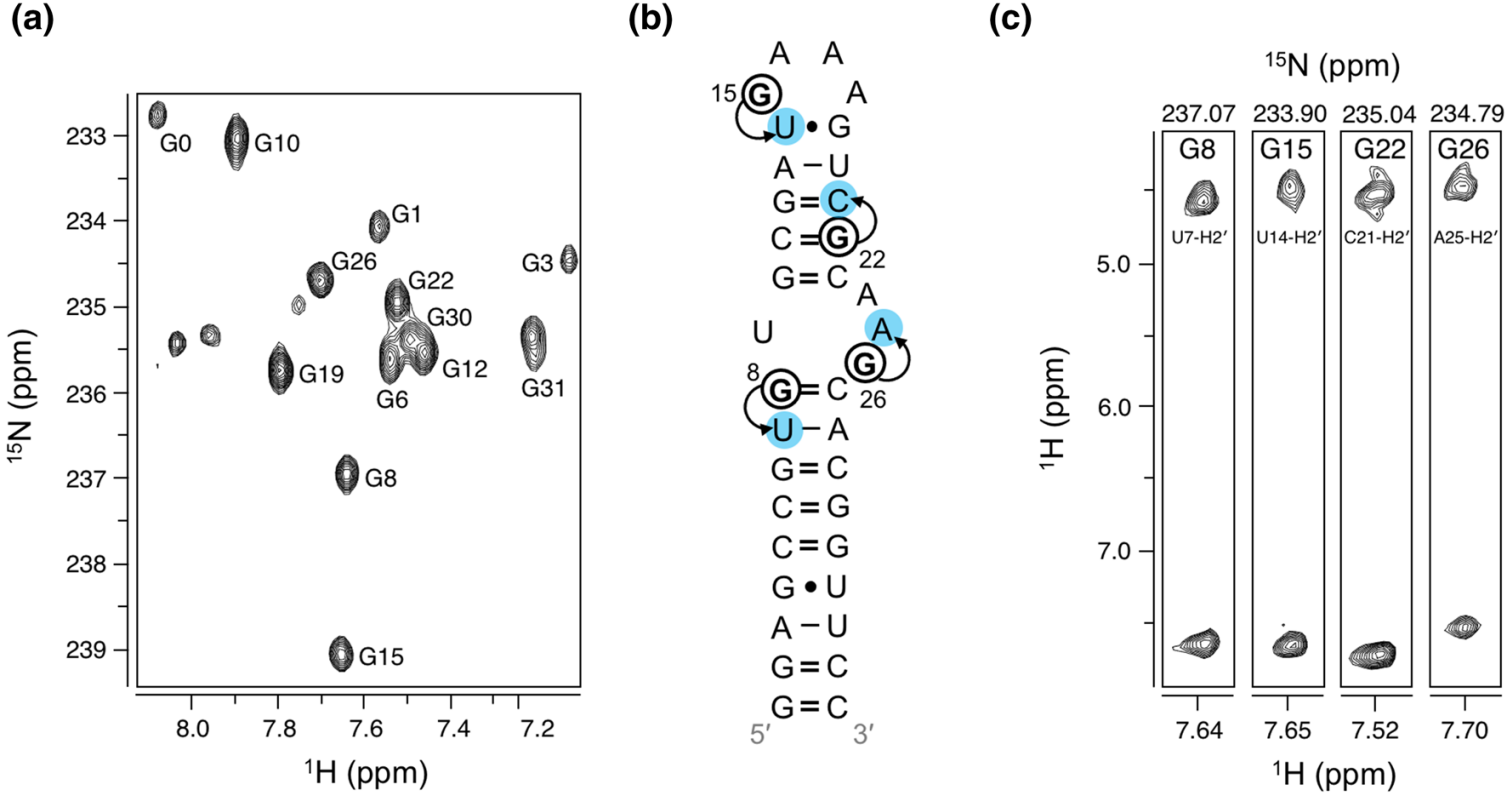
HSQC and ^15^N-edited NOESY HSQC experiments in **4a**-labeled domain 5 RNA. **a** 2D ^1^H,^15^N HSQC spectra showing H8-N7 resonances. **b** Representation of NOE contacts of H8 protons to H2′ protons for select nucleotides G8, G15, G22, and G26. **c** 2D ^1^H,^1^H slice along a single ^15^N frequency for the same select nucleotides shown in **b**. All spectra are annotated with RNA resonance assignments

As a first application, we employed a two-bond (^2^*J*_H8N7_) 2D heteronuclear single quantum coherence (HSQC) experiment on **4a**-labeled domain 5 RNA. We obtained a well-resolved 2D spectrum, showing all 13 guanosine H8-N7 resonances (Fig. 2a). A necessary NMR parameter for structure determination is proton–proton distances, which is provided by nuclear Overhauser effect spectroscopy (NOESY) experiments. While these data are informative, crowded proton–proton NOEs can be resolved into a third dimension with ^13^C- or ^15^N-editing. As a second application, we employed a ^15^N-edited 3D NOESY HSQC experiment on **4a**-labeled domain 5 to reveal all protons within ~ 5 Å of guanosine H8 [38]. In A-helical RNA, guanosine H8 protons show strong NOE cross-peaks to 5′-neighboring H2′ protons (Fig. 2b), which are traditionally difficult to assign due to severe overlap with other ribose protons (i.e., H3′, H4′, H5′, and H5″) [37–39]. We therefore used the chemical shift of guanosine N7 to resolve NOE cross-peaks of H2′ protons to H8. Examples of cross-peaks are shown for helical residues G8 and G22, as well as residues G15 and G26 from the apical loop and internal bulge, respectively (Fig. 2c).

**Fig. 3:**
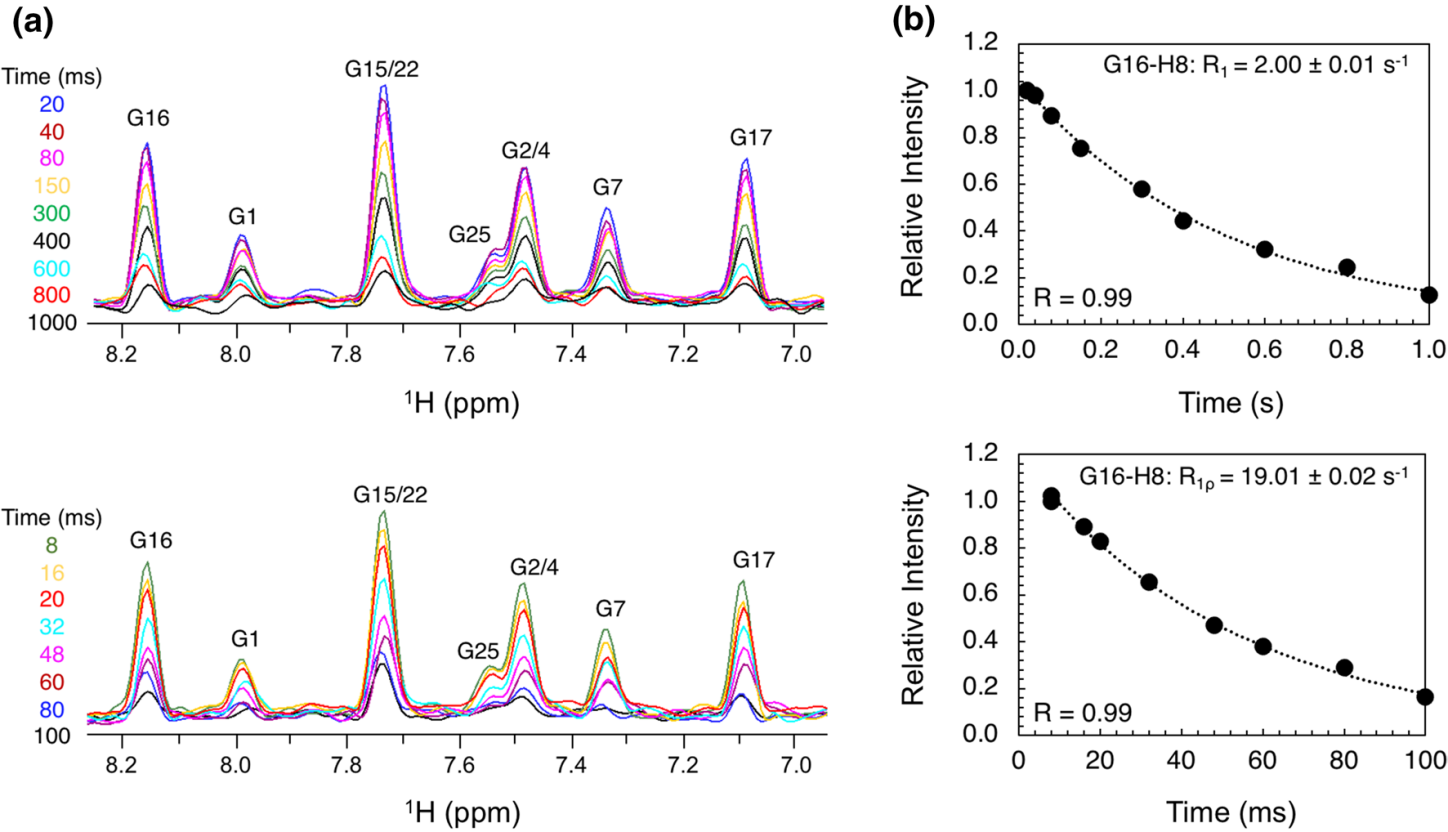
Dynamics measurements in **4b**-labeled A-site RNA. **a** Pseudo-2D HSQC-based spectra of *R*_1_ (top) and *R*_1ρ_ (bottom) experiments, with all relaxation delays shown. **b** Representative *R*_1_ (top) and *R*_1ρ_ (bottom) decay curves are shown for G16-H8. Extracted rate and curve fit are shown

As a final application, we probed the dynamics of **4b**-labeled A-site RNA. Two common relaxation parameters in biomolecules are the longitudinal (*R*_1_) and transverse (*R*_2_) relaxation rates [40, 41]. While *R*_1_ measures the rate of recovery of the *z*-magnetization to equilibrium, *R*_2_ reports on the rate of the decay of *x*- and *y*-magnetization [40]. An alternative method to obtain *R*_2_ is a transverse rotating-frame (*R*_1ρ_) experiment wherein magnetization is aligned along an effective field whose direction is dependent upon the power of the radio frequency (RF) field and its offset [42]. We therefore employed pseudo-2D HSQC-based experiments to determine *R*_1_ and *R*_1ρ_ relaxation rates of guanosine H8 protons (Fig. 3a). We obtained rates for 5 of the 9 guanosines in A-site, with *R*_1_ and *R*_1ρ_ values ranging from 1.75 to 2.00/s and 15.26 to 21.25/s, respectively. Interestingly, G16 showed the highest *R*_1_ and lowest *R*_1ρ_, indicative of increased flexibility, suggesting the G:C base pair preceding the tetraloop is not stable. These data contribute to our understanding of the dynamic motions within the A-site RNA [43, 44].

## Conclusion

We report the synthesis of atom-specifically labeled [7-^15^N]-GTPs for use in in vitro transcription to make RNA for NMR analysis. Our synthetic routes include a combined chemical and enzymatic approach, using inexpensive commercially available starting materials. To demonstrate the utility of our new labels, we introduced them into two RNAs via in vitro transcription to permit straightforward NMR structure and dynamics measurements. We anticipate these labels will aid efforts to probe in greater detail the structure, interactions, and dynamics of biologically and medically RNAs.

## Experimental

Commercially available reagents were used without further purification unless explicitly stated. All reagents used for the synthesis of **3** were purchased from Sigma-Aldrich. All reactions were carried out under nitrogen or argon atmosphere. All non-commercially available enzymes were expressed and purified in-house using established methods [28]. DNA templates for in vitro transcription of RNAs were purchased from Integrated DNA Technologies (IDT, Coralville, IA) and used without further purification. Chromatographic purification was carried out using boronate affinity resin with eluent specified. ^1^H NMR spectra were recorded on a Bruker Avance I 300 MHz or a Bruker Avance Neo 400 MHz spectrometer, ^13^C NMR spectra were recorded on a Bruker Avance III 500 MHz spectrometer, ^15^N NMR spectra were recorded on a Bruker Avance III 700 MHz spectrometer, and ^31^P NMR spectra were recorded on an Avance III Bruker 800 MHz spectrometer with a triple resonance cryogenic probe. Samples were maintained at a temperature of 25 °C. All NMR experiments for RNA were performed in D_2_O and all chemical shifts were reported in ppm (parts per million). All RNA spectra were referenced to DSS (4,4-dimethyl-4-silapentane-1-sulfonic acid). Nitrogen-15 and Carbon-13 chemical shifts were indirectly referenced using the ratio of the gyromagnetic ratios of proton to ^15^N (0.101329118) and ^13^C (0.251449530), respectively [45, 46]. All NMR experiments for compounds **1**, **2**, and **3** were performed in DMSO-*d*_*6*_ or D_2_O. The chemical shifts of compounds **1**, **2**, and **3** were referenced to the residual protonated solvent signal of DMSO-*d*_*6*_ (2.5 ppm) or D_2_O (HDO 4.7 ppm) as previously reported [47]. The ^15^N dimension was referenced using the liquid ammonia referencing implemented in the Topspin software suite.

### 2,6-Diamino-5-[nitroso-^15^N]pyrimidin-4-ol (1, C_4_H_5_N_4_^15^NO_2_)

To begin the chemical synthesis of [7-^15^N]-guanine, 3.65 g 2,6-diaminopyrimidin-4-ol (29.00 mmol) and 2.30 g ^15^N-labeled sodium nitrite (33.30 mmol) were dissolved in 40 cm^3^ of 3 M sodium hydroxide. The homogeneous solution obtained from the previous step was then added dropwise to 50 cm^3^ glacial acetic acid, while stirring and cooling on ice, and gave rise to a pink precipitate. The precipitate was isolated by centrifugation, washed with cold water, ethanol, and diethyl ether, and dried in high vacuum (1 × 10^–2^ mbar) on a vacuum line for 8 h to give pure compound **1**. Yield: 3.55 g (79%); ^1^H NMR (300 MHz, DMSO-*d*_*6*_): *δ* = 7.34 (br s, 4H) ppm; HR-MS (ESI–MS): *m/z* calculated for C_4_H_5_N_4_^15^N_1_O_2_ + H^+^ 157.0486 Da, found 157.0486 Da.

### 2,6-Diamino-5-[amino-^15^N]pyrimidin-4-ol (2, C_4_H_7_N_4_^15^NO)

After vacuum drying, compound **1** was resuspended in 80 cm^3^ boiling water and 9.96 g sodium dithionite (57.23 mmol) was added in several portions to give a pink suspension. The reaction was kept at 100 °C and the pink suspension became yellow in color. The mixture was then cooled in an ice bath for 30 min. The yellow solid was collected by filtration and then resuspended in 65 cm^3^ of 2 M sulfuric acid, heated to 80 °C for 20 min, and cooled on ice to give a sulfate salt. This salt was collected by filtration, washed with ethanol, and dried in high vacuum (1 × 10^–2^ mbar) on a vacuum line for 12 h to give pure compound **2**. Yield: 5.45 g (22.8 mmol); ^1^H NMR (400 MHz, DMSO-*d*_*6*_): *δ* = 8.94 (br s, 2H), 6.46 (br s, 2H), 6.34 (br s, 2H) ppm; HR-MS (ESI–MS): *m/z* calculated for C_4_H_7_N_4_^15^N_1_O_1_ + H^+^ 143.0694 Da, found 143.0693 Da.

### [7-^15^N]-2-Amino-1*H*-purin-6(9*H*)-one (3, C_5_H_5_N_4_^15^NO)

Compound **2** was dissolved in 30 cm^3^ formamide and formic acid and then refluxed at 110 °C and 180 °C for 1 and 2 h, respectively, to form a yellow solution. After cooling on ice for 30 min, a milky precipitate formed. The precipitate was filtered, rinsed with water, and then dissolved in 35 cm^3^ 10% aqueous sodium hydroxide. Crude product **3** was precipitated by neutralizing the solution on ice with formic acid. A fine, yellowish powder of crude compound **3** was obtained by filtering the precipitate, which was then washed with water and ethanol to remove any residual formamide contaminant and dried in high vacuum (1 × 10^–2^ mbar) on a vacuum line for 6 h to yield pure compound **3**. Yield: 2.85 g (83%); ^1^H NMR (400 MHz, DMSO-*d*_*6*_): *δ* = 8.89 (d, ^2^*J*_NH_ = 5.92 Hz, 1H) ppm; ^15^N NMR (60 MHz, DMSO-*d*_*6*_): *δ* = 241.39 (^15^N7) ppm; ^1^H NMR (700 MHz, 10 mM NaOD in D_2_O): *δ* = 7.49 (d, ^2^*J*_NH_ = 12 Hz, 1H) ppm; ^13^C NMR (176 MHz, 10 mM NaOD in D_2_O): *δ* = 167.53 (C4), 160.94 (C6), 159.11 (C2), 148.56 (C8), 118.24 (C5) ppm; ^15^N NMR (70 MHz, 10 mM NaOD in D_2_O): *δ* = 221.83 (^15^N7) ppm; HR-MS (ESI–MS): *m/z* calculated for C_5_H_5_N_4_^15^N_1_O_1_ + H^+^ 153.0537 Da, found 153.0537 Da.

### [7-^15^N]-[[[[(2*R*,3*S*,4*R*,5*R*)-5-(2-Amino-6-oxo-1,6-dihydro-9*H*-purin-9-yl)-3,4-dihydroxytetrahydrofuran-2-yl]methoxy]oxidophosphoryl]oxidophosphoryl]phosphonate (4a, C_10_H_12_N_4_^15^NO_12_P_3_^4−^)

The one-pot reaction was carried out in a 50 cm^3^ Falcon tube. The reaction mixture consisted of 9.1 mg compound **3** (6.0 mM), fully protonated [^1^H_6_]-d-ribose (6 mM), 10 mM MgCl_2_, deoxyadenosine-5′-triphosphate (0.5 mM), Bovine serum albumin (0.1 mg/cm^3^), dithiothreitol (10 mM), potassium chloride (100 mM), sodium phosphate monobasic (9.4 mM, pH 6.5), sodium phosphate dibasic (40 mM, pH 6.5), RK (1.0 × 10^–5^ U/mm^3^), PRPPS (1.0 × 10^–5^ U/mm^3^), XGPRT (0.01 mg/cm^3^), GK (0.01 mg/cm^3^), MK (0.01 U/mm^3^), and CK (0.05 mg/cm^3^). The reaction was incubated at 37 °C for 12 h. After confirming successful triphosphate formation by ^31^P NMR, crude compound **4a** was purified by boronate affinity chromatography (eluent A: 1 M triethylamine pH 9; eluent B: acidified water pH 4), lyophilized to a powder, and resuspended in Ultrapure water. Yield: 6.8 mg (~ 90%).

### [1′,2′,3′,4′,5′,5″-^2^H_6_,7-^15^N]-[[[[(2*R*,3*S*,4*R*,5*R*)-5-(2-Amino-6-oxo-1,6-dihydro-9*H*-purin-9-yl)-3,4-dihydroxytetrahydrofuran-2-yl]methoxy]oxidophosphoryl]oxidophosphoryl]phosphonate (4b, C_10_H_6_^2^H_6_N_4_^15^NO_12_P_3_^4−^)

The one-pot reaction was carried out in a 50 cm^3^ Falcon tube. The reaction mixture consisted of 9.1 mg compound **3** (6.0 mM), fully deuterated [^2^H_6_]-d-ribose (6 mM), 10 mM MgCl_2_, deoxyadenosine-5′-triphosphate (0.5 mM), Bovine serum albumin (0.1 mg/cm^3^), dithiothreitol (10 mM), potassium chloride (100 mM), sodium phosphate monobasic (9.4 mM, pH 6.5), sodium phosphate dibasic (40 mM, pH 6.5), RK (1.0 × 10^–5^ U/mm^3^), PRPPS (1.0 × 10^–5^ U/mm^3^), XGPRT (0.01 mg/cm^3^), GK (0.01 mg/cm^3^), MK (0.01 U/mm^3^), and CK (0.05 mg/cm^3^). The reaction was incubated at 37 °C for 12 h. After confirming successful triphosphate formation by ^31^P NMR, crude compound **4b** was purified by boronate affinity chromatography (eluent A: 1 M triethylamine pH 9; eluent B: acidified water pH 4), lyophilized to a powder, and resuspended in ultrapure water. Yield: 2.5 mg (~ 33%). The low yield of this reaction compared to that of **4a** was due to complications with purification, not with conversion of **3** to **4b**.

### RNA preparation

RNAs were synthesized via in vitro transcription. The reactions were carried out in a 10 cm^3^ reaction volume at 37 °C. The reactions consisted of transcription buffer (40 mM Tris–HCl pH 8.0, 1 mM spermidine and 0.01% Triton-10), 0.3 µM single strand DNA template, 80 mg/cm^3^ PEG, 1 mM DTT, 2 U/mm^3^ thermostable inorganic pyrophosphatase, 10 mg/cm^3^ T7 RNA polymerase, a total of 5 mM rNTPs (1.25 mM each of unlabeled ATP, CTP, UTP, and either **4a** or **4b**), and 7.5 mM MgCl_2_. The concentrations of rNTPs and MgCl_2_ were chosen following optimization at small (50 mm^3^) and mid-scale (500 mm^3^) reactions. All reactions were quenched after 3 h by adding 0.5 mM EDTA. Following transcription, the RNAs were purified via preparative polyacrylamide (12%) gel electrophoresis, electroeluted, and exchanged into deionized water. Then NMR buffer (A-site: 50 mM NaCl, 50 mM Na_3_PO_4_, 100 mM EDTA, 0.02% NaN_3_, 0.1 mM DSS, 10% D_2_O; domain 5: 100 mM KCl, 10 mM K_3_PO_4_, 0.02% NaN_3_, 0.1 mM DSS, 10% D_2_O) was added and the samples were lyophilized and resuspended in D_2_O (99.9%). The concentration of the purified samples ranged between 0.3 and 0.5 mM in 300 mm^3^.

### NMR experiments

All experiments on **4a**- and **4b**-labeled samples were carried out at 25 °C on a Bruker 600 MHz magnet Avance III spectrometer with a TXI triple resonance probe in 5 mM Bruker optimized Shigemi NMR tubes. 2D-^15^N Selective Optimized Flip Angle Short Transient (SOFAST) [48] HSQC spectrum of compound **3** was obtained with 16 scans and 1024 × 64 complex points in the ^1^H and ^15^N dimensions, respectively. An INEPT delay of 31.25 ms (a coupling constant of 8 Hz) was used for coherence transfer. For the ^1^H dimension, the spectral width was set to 20 ppm and the carrier to 12 ppm. For the ^15^N dimension, the spectral width was set to 5 ppm and the carrier to 240 ppm. The two-bond (^2^*J*_H8N7_) 2D HSQC experiment was performed with 128 scans and 100 and 1024 complex points in the ^15^N and ^1^H dimensions, respectively. The ^15^N and ^1^H carrier frequencies were set to 235.5 ppm and 4.7 ppm, respectively; the spectral width for the ^15^N and ^1^H dimensions were set to 9 and 12 ppm, respectively. The ^15^N-edited 3D NOESY HSQC experiment was carried out on a 600 MHz magnet Avance III spectrometer with a TXI triple resonance probe using previously described pulse sequences [38]. For each 3D data set, 200 × 128 complex points were used for the indirect ^1^H and ^15^N dimensions along with 64 transients and 10% non-uniform sine-weighted Poisson-gap sampling [49]. The spectral width of the ^15^N and ^1^H dimensions were set to 5.2 and 10.0 ppm, respectively; the carrier was set to 235.1 and 4.7 ppm, for the ^15^N and ^1^H dimensions, respectively.

*R*_1_ and *R*_1ρ_ relaxation rates on the guanosine-N7 nitrogen atoms were measured using a pseudo-2D HSQC detected experiments. These experiments were carried out with 16 scans and 1024 time domain points in the ^1^H dimension, using a spectral width and carrier of 16 and 4.7 ppm, respectively. For the *R*_1_ experiments, delay times of 0.02 (X2), 0.04, 0.08, 0.15, 0.30, 0.40, 0.60, 0.80, and 1.00 s were used. For the *R*_1ρ_ experiments, delay times of 8 (X2), 16, 20, 32, 48, 60, 80, and 100 ms were used. The strength of spin-lock field (*ω*_1_) was 1.9 kHz. Calibration of the spin-lock field was carried out as previously described [50]. *R*_1_ and *R*_1ρ_ relaxation rates were determined by fitting intensities to a monoexponential decay and errors were estimated from duplicated delay points. All NMR data were collected at 25 °C with a recycle delay of 1.5 s and analyzed using TopSpin 4.0 and NMRViewJ [51].

## Supplementary Information

Below is the link to the electronic supplementary material.

Supplementary file1 (DOCX 16252 kb)
