# Supplementary material for: Synthesis of [7-15N]-GTPs for RNA structure and dynamics by NMR spectroscopy
Source: Monatsh Chem. 2022 Feb 26;153(3):293–9. doi: 10.1007/s00706-022-02892-1 (PMC8948113; doi:10.1007/s00706-022-02892-1)

**Synthesis of 7-^15^N-GTPs for RNA structure and dynamics by NMR spectroscopy**

**Kehinde M. Taiwo^1^ ● Lukasz T. Olenginski^1^ ● Felix Nußbaumer ^2^ ● Hyeyeon Nam ^1,#^ ● Stefan Hilber^2^ ● Christoph Kreutz ^2^● T. Kwaku Dayie ^1^**

1. Center for Biomolecular Structure and Organization, Department of Chemistry and Biochemistry, University of Maryland, College Park, MD 20742, United States.
2. Institute of Organic Chemistry and Center for Molecular Biosciences Innsbruck, University of Innsbruck, Innrain 80/82, 6020 Innsbruck, Austria.

^#^Current Address: Center for Cancer Research, National Cancer Institute, Frederick, MD 21702, United States.

**Supplementary Table of Contents:**

**Figure S1.** ^1^H NMR of compound **1**.

**Figure S2.** ^1^H NMR of compound **2**.

**Figure S3.** ^1^H and ^13^C NMR of compound **3**.

**Figure S4.** ^15^N NMR and ^1^H-^15^N long range HSQC of compound **3**.

**Figure S5.** ESI-MS analysis of compounds **1** to **3**.

**Figure S6.** Representative ^31^P NMR of GTP reactions shown for compound **4a**.

**Figure S1.** ^1^H NMR (300 MHz, DMSO-d_6_) of compound **1**.


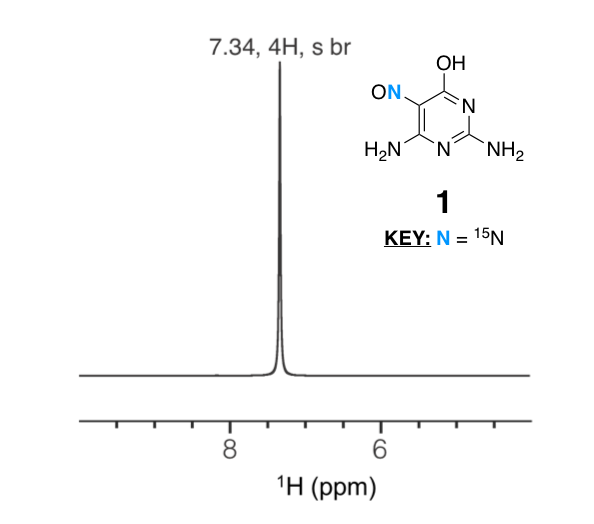


**Figure S2.** ^1^H NMR (400 MHz, DMSO-d_6_) of compound **2**.

**
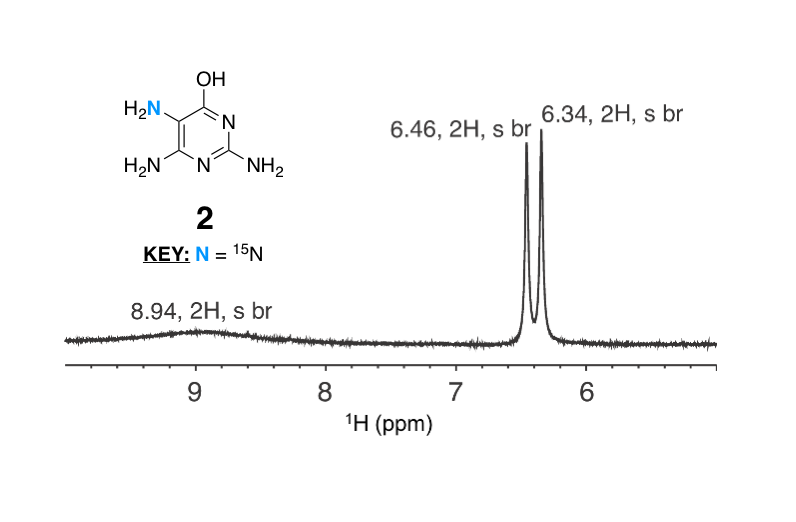
**

**Figure S3.** **A)** ^1^H NMR (700 MHz) and **B)** ^13^C NMR (176 MHz, 10 mM NaOD in D_2_O) of compound **3**.


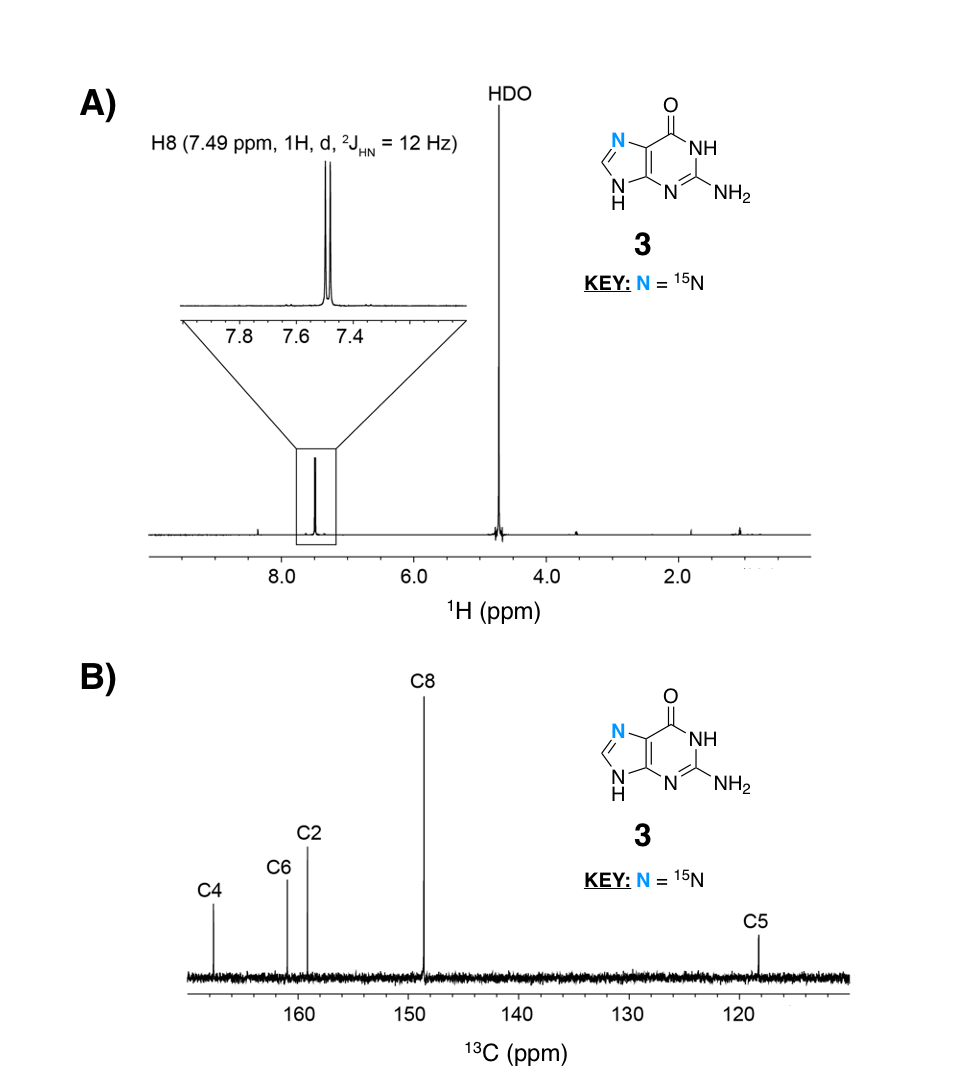


**Figure S4. A)** ^15^N NMR (70 MHz) and **B)** ^1^H-^15^N long range HSQC (700/70 MHz, 10 mM NaOD in D_2_O) of compound **3**.


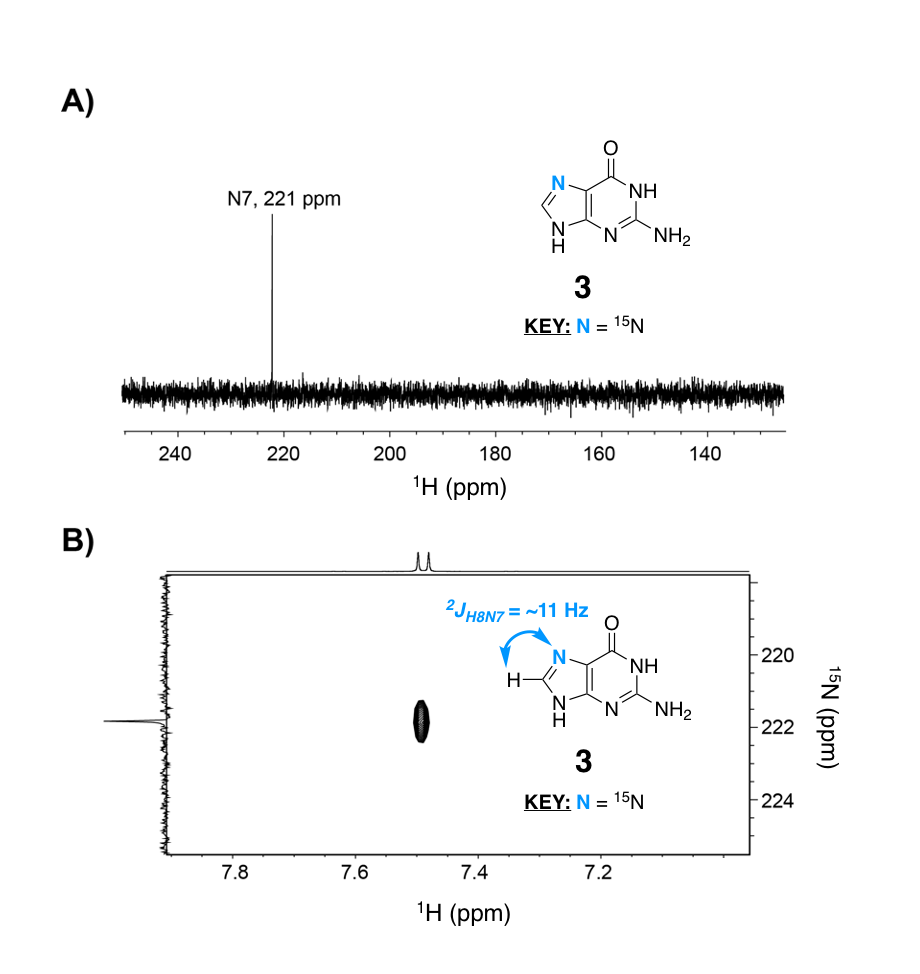


**Figure S5.** Comparison of experimental and calculated ESI mass spectra of compounds **1 (A)**, **2 (B)** and **3 (C)**.


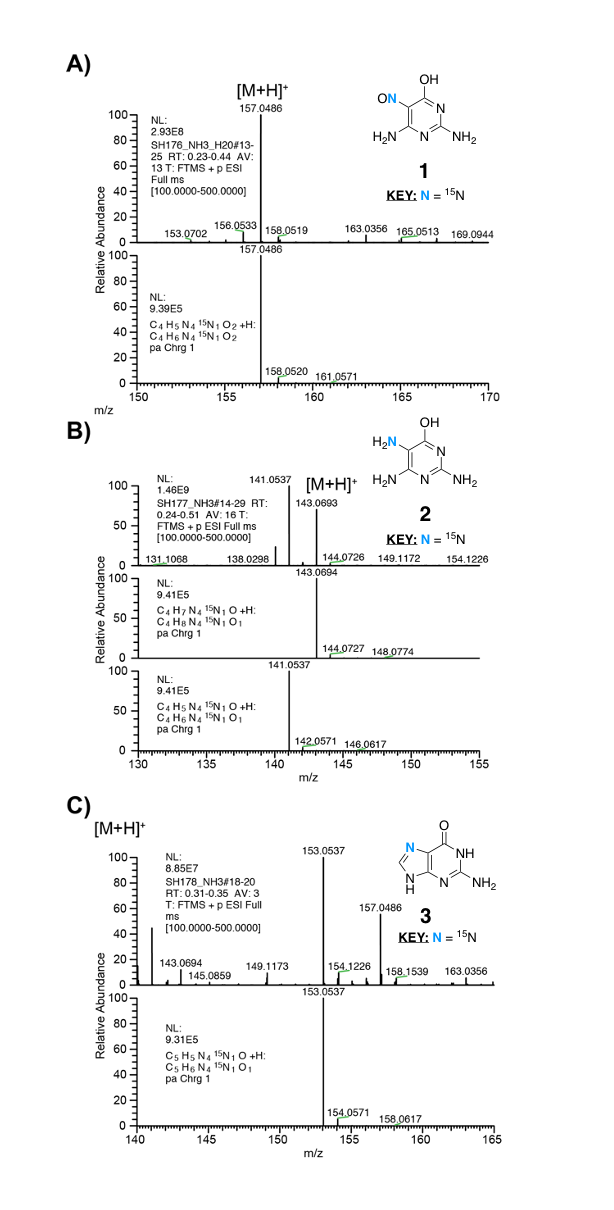


**Figure S6.** Representative ^31^P NMR of GTP reactions shown for compound **4a**. Peaks belonging to the α, β, and γ phosphate peaks are labeled in the spectrum and shown on the structure of compound **4a**.


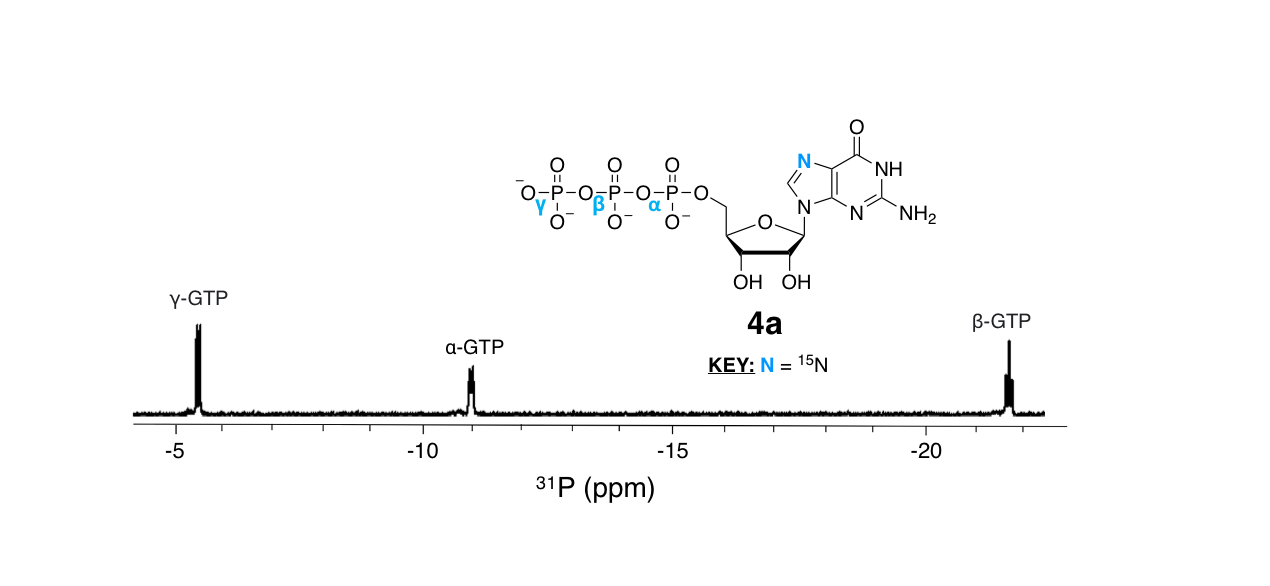

Supplement: Supplementary file 1 — Supplementary file1 (DOCX 16252 kb) [file 706_2022_2892_MOESM1_ESM.docx]
